# Stereotactic radiotherapy of radiation-induced meningioma previously irradiated retrobulbar for Graves' ophthalmopathy: A case report

**DOI:** 10.1016/j.radcr.2023.11.052

**Published:** 2023-12-15

**Authors:** Nao Tachizawa, Takeshi Kondoh, Masahiro Sugihara, Hirotomo Tanaka, Yoshiyuki Takaishi, Hidehito Kimura, Takashi Sasayama

**Affiliations:** a Department of Neurosurgery, Shinsuma General Hospital, Kobe, Japan; b Department of Neurosurgery, Kobe University Graduate School of Medicine, Kobe, Japan

**Keywords:** Gamma Knife radiosurgery, Radiation-induced tumor, Atypical meningioma, Graves' ophthalmopathy

## Abstract

A 69-year-old woman was diagnosed with an asymptomatic intracranial tumor nine years ago and has been followed with annual MR imaging studies. Two years ago, the tumor had grown in size, requiring treatment. She experienced ophthalmopathy due to hyperthyroidism 27 years ago and was treated with 20 Gy in 10 fractions using parallel opposed beams to her bilateral posterior eyeballs, supplemented with steroid pulse therapy. The tumor originated in the medial aspect of the right sphenoid border and compressed the temporal lobe, while bone infiltration was observed, partially extending to the soft tissue outside the maxillary sinus. The tumor was removed by craniotomy. The pathological diagnosis was atypical meningioma (WHO grade II). Four months postsurgery, the resection cavity's tumor exhibited growth inclination, necessitating Gamma Knife radiosurgery. Radiation planning was executed at a marginal tumor dose of 30 Gy in 5 fractions. Since the optic nerve had been previously exposed to radiation, a plan was devised to minimize radiation exposure. The dose on the optic nerve was limited to 6.9 Gy in 5 fractions. She did not experience any visual or visual field disruptions postradiation. This is a case of radiation-induced meningioma resulting from radiation therapy for Graves' ophthalmopathy and is the first reported case of a grade II meningioma. The patient's condition calls for adjuvant radiation therapy following surgical removal. Accordingly, a radiation treatment plan that safeguards the optic nerve, which was previously exposed to radiation, was deemed indispensable.

## Introduction

Graves' ophthalmopathy may lead to notable and escalating impairments to vision [1]. Emergency treatment for this condition has involved radiation therapy to the orbital region, alongside steroid pulse therapy [2–6]. Previously, opposing portal irradiation was used for conventional irradiation [5,6]. However, advancements in radiation therapy approaches now enable irradiation of solely the orbital contents [2–4].

In irradiation utilizing 2 opposing radiation sources, there is a potential risk of exposing normal bone and soft tissue, which could lead to adverse effects. The literature reports 1 case of radiation-induced meningioma (grade I) which was treated by surgery alone [6]. In addition to the risk of radiation-induced tumors, high-dose exposure of the intraorbital optic nerve is unavoidable with parallel opposed beams. With modern software, it is possible to reduce the dose to adjacent organs-at-risk (OAR) to an extremely low level, precise radiotherapy planning is crucial when treating a patient requiring radiotherapy near the orbit for another illness following the resolution of Graves' ophthalmopathy.

In this study, we report a case of radiation-induced meningioma that was treated 27 years after radiotherapy for Graves' ophthalmopathy. This is only the second report of such a case in the literature. Additionally, our case is the first to receive postoperative radiotherapy, and we highlight the importance of attention to reducing optic nerve exposure during treatment.

## Case presentation

**Fig. 1:**
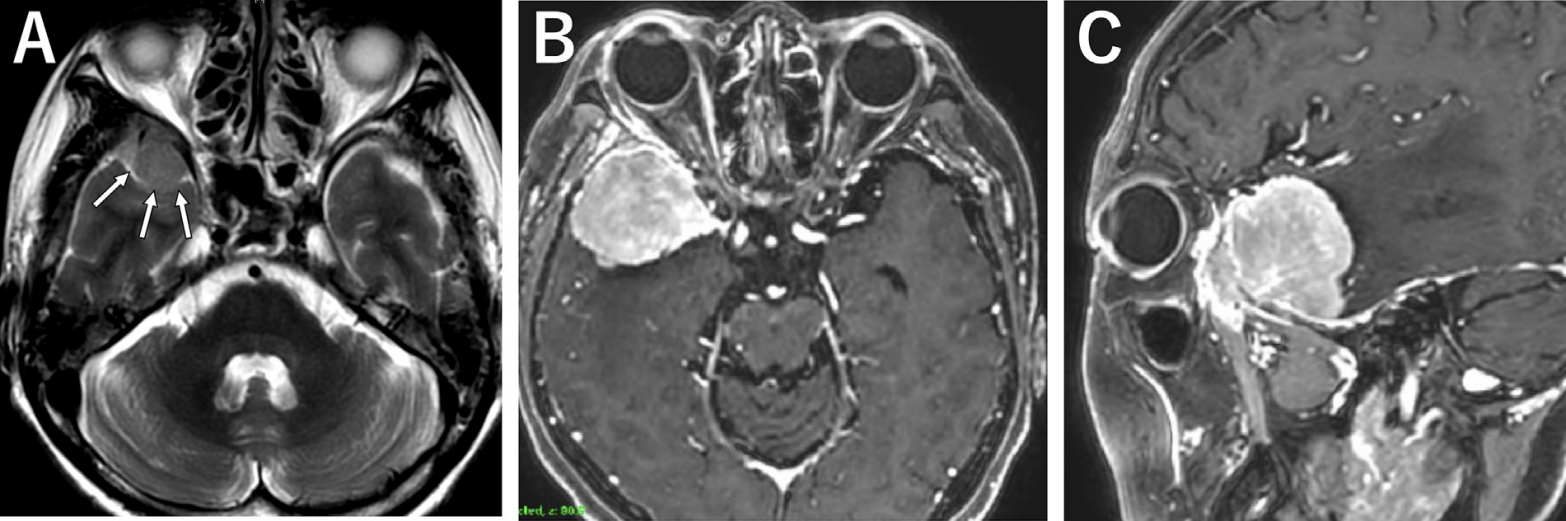
Axial plain T2- weighted MR image 6 years before surgery showing a meningioma at the right-sided sphenoidal wing measuring 15 × 13 × 11 mm (arrows). (A. Contrast-enhancement T1-weighted MR images of axial (B) and sagittal (C) view at the time of surgery showing the tumor measuring 38 × 38 × 41 mm.

A 69-year-old woman was diagnosed with an asymptomatic intracranial tumor at the age of 60. The tumor was 15 × 13 × 11 mm in diameter and discovered during a head MRI scan used to treat low intracranial pressure syndrome. Several blood patch treatments successfully cured the syndrome (Fig. 1A). At age 67, a follow-up MRI revealed that the tumor had significantly increased in size. At the age of 68 and 69, MRI scans indicated a significant increase in tumor size without any symptoms such as headache, diplopia, or visual disturbances, leading to the patient's referral to our hospital for treatment.

At 42 years old, the patient experienced hyperthyroidism and Graves' ophthalmopathy, leading to diplopia. After 6 months of Mercazole treatment, the patient received radiation therapy targeting the posterior region of the orbital eye. Steroid pulse therapy was also administered due to the heightened danger of vision loss and progressive protrusion. The patient received radiation therapy (20 Gy/10 Fr) to the orbital retro-orbital region along with steroid pulse therapy. The protrusion was promptly rectified after the treatment, and there was no reappearance or visual field damage observed henceforth. There was no chart available for the 2 opposing irradiations. Based on the patient record, the irradiation method used was the standard method at that time, involving a basic skull scan to verify the lesion location. A CT scan taken during treatment revealed no neoplastic lesions.

**Fig. 2:**
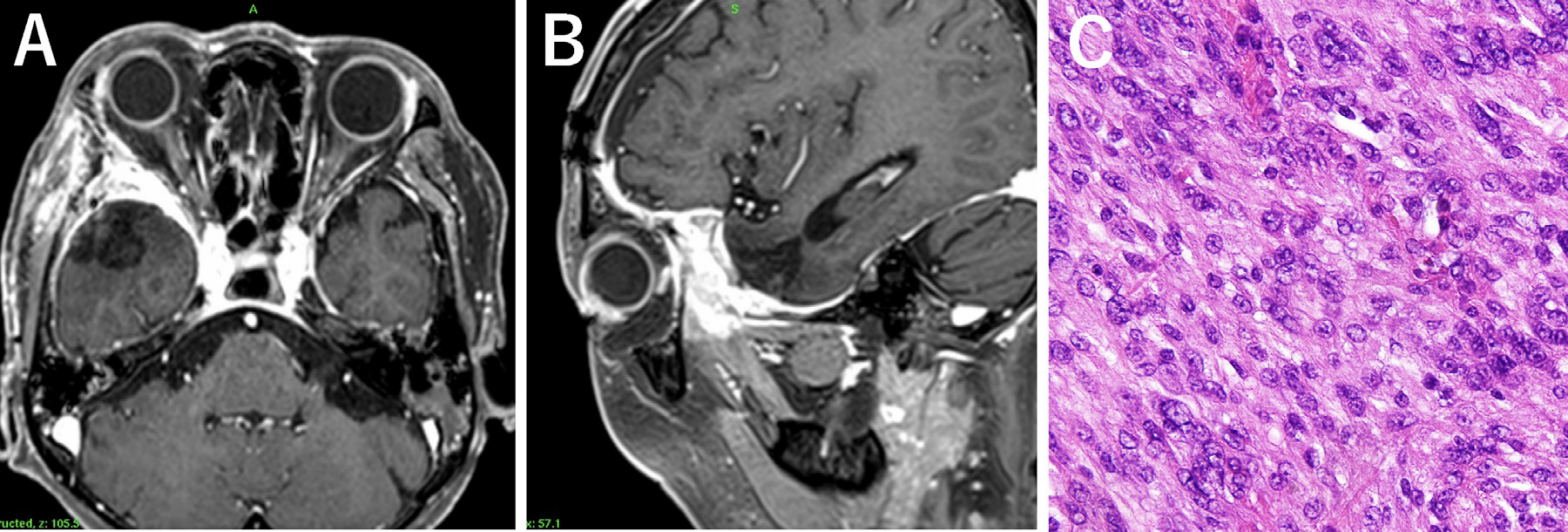
Contrast-enhancement T1-weighted MR images of axial (A) and sagittal (B) view immediately after surgery. The histopathologic examination demonstrated an atypical meningioma (hematoxylin-eosin staining) (C).

During the consultation, an MRI scan revealed a 38 × 38 × 41 mm tumor on the right sphenoid margin (Figs. 1B and C). The gadolinium contrast medium showed a marked contrast of the tumor, with a volume of 31.3 cc. Surrounding cerebral edema was moderate. The tumor attachment site was lateral to the sphenoid margin. A craniotomy was performed, which revealed that the tumor tissue had destroyed the dura mater and invaded the sphenoidal bone marrow. The tumor was removed from the intracranial skull, including the intraosseous tumor (Figs. 2A and B). The histopathologic examination revealed an atypical meningioma (WHO grade II) (Fig. 2C). Polygonal and spindle-shaped cells with sporangia were observed proliferating in patternless bundles and sheets, as well as spiral structures and intranuclear inclusion bodies. Enlarged nuclei and irregularly sized nuclei were also seen in some areas. Immunostaining revealed positivity for EMA and a Ki-67 of 10%, without apparent necrosis. GFAP staining indicated infiltration into the brain parenchyma.

**Fig. 3:**
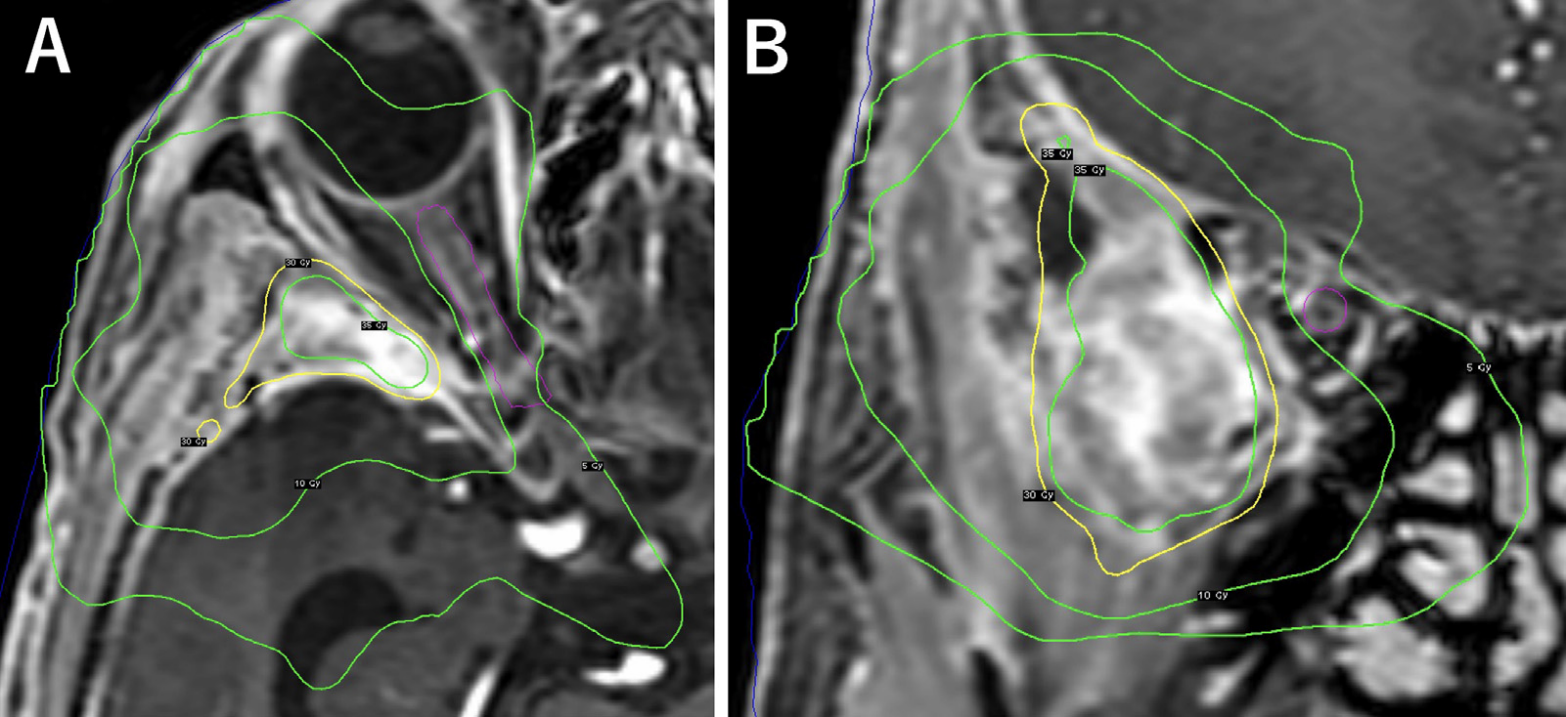
Planed dose distributions on contrast-enhancement T1-weighted axial MR image (A) and coronal MR image (B). Isodose lines indicate from 5 Gy to 40 Gy. The right optic nerve is set as organs-at-risk (pink line).

A postoperative MRI 4 months later showed residual tumor growth in both the soft tissue of the lateral maxillary sinus and the extraction cavity, prompting gamma knife radiosurgery for the patient. Frameless stereotactic radiotherapy using the Gamma Knife Icon was performed, in which a 3-point thermoplastic mask was created for immobilization. The clinical target volume (CTV) was 5.0 cc. The planning target was generated to cover not only the tumor but also the sphenoidal bone edge and surrounding soft tissue of extracranial lesion. The planning target volume (PTV) was 11.1 cc. The marginal dose at the 64% isodose line was 30 Gy in 5 fractions (Figs. 3A and B). In the present case, residual tumor was located an acceptable distance from the optic apparatus but since it had received 20 Gy irradiation for Grave's ophthalmopathy in the past, fractionated radiosurgery was considered to be safer for the optic apparatus than single large-dose radiosurgery, without compromising the tumor control rate. The optic nerve was set as organs-at-risk considering the maximum dose was 9.3 Gy and the mean dose was 6.9 Gy. At 8 months post-treatment, there was no impairment of visual field due to gamma knife radiosurgery and the patient is doing well with no re-growth of the tumor.

## Discussion

### Graves' ophthalmopathy and retrobulbar radiation

Graves' ophthalmopathy affects up to around 50% of patients within 12-18 months of thyroid disease onset. In 2022, a consensus statement by the American Thyroid Association and the European Thyroid Association has been published [1]. During the active disease phase, accumulation of hydrophilic glycosaminoglycans, interstitial edema, increased adipogenesis, and lymphocyte infiltration of orbital tissues are all characteristic findings. In addition to a formal ophthalmological evaluation, imaging studies reveal extraocular muscle enlargement leading to periorbital soft tissue congestion, ocular motility restriction, and optic nerve compression resulting in dysthyroid optic neuropathy.

Regarding the treatment of Graves' ophthalmopathy, glucocorticoids have been extensively studied and utilized for over 60 years. If patients are unresponsive to glucocorticoids, Rituximab, Tocilizumab, and Teprotumumab may be prescribed, followed by radiation therapy [1]. Radiation therapy inhibits or depletes lymphocytes and fibrocytes in the affected orbital tissue and has been a treatment option for Graves' ophthalmopathy for over 70 years. The effectiveness of radiation therapy in treating Graves' ophthalmopathy has shown variability in prior randomized studies. While it may not be effective for late-stage or inactive disease, it is a preferred treatment for patients with active moderate-to-severe Graves' ophthalmopathy. It is generally considered inappropriate for individuals under 35 years old, due to the risk of radiation-related second malignancies as a late side effect. Additionally, caution must be taken when administering the treatment to patients with diabetes, as it may lead to retinopathy.

Until approximately 2010, radiotherapy was conducted using bilateral retrobulbar irradiation at a total dose of 20 Gy administered with a linear accelerator over a 2-week period, with daily fractions of 2 Gy given 5 times per week, as done in the present case [5,6]. Regarding complications of conventional treatment, only 1 case of radiation-induced meningioma has been reported [6]. Like in the present case, the origin of attachment was on the medial side of the sphenoid ridge, and it was surgically removed. The pathological diagnosis was WHO grade I meningioma, which had moderate cellularity with few areas of mitoses.

### Radiation-induced meningioma and stereotactic radiation

Between 1984 and 2010, a single institute reported that the incidence of radiation-induced meningioma after an average follow-up of 14 years with at least 3 years of follow-up after irradiation was 0.17% [6]. Another study of bilateral retrobulbar irradiation with an average follow-up of 11.3 years between 1982 and 1993, found that retinopathy was present in 15% of patients but no radiation-induced tumor observed [5]. Diabetes was associated with both possible and definite retinopathy, with a relative risk of 21, indicating that orbital irradiation for Graves’ ophthalmopathy is a safe treatment option, except for diabetic patients who may experience adverse effects.

More recently, since about 2010, treatment with a 3D conformal technique has been reported instead of bilateral retrobulbar irradiation [3,4]. Radiation therapy is planned with a 3D conformal technique delivered. The CTV encompassed the bilateral orbits from the apex of the sphenoid sinus posteriorly to the fleshy cantus anteriorly and from the roof of the orbit superiorly to its floor inferiorly. A margin of 5-8 mm in all directions was added to the CTV to generate the PTV. These studies using 20 Gy in 10 fractions, with short follow-up periods ranging from a few years to within 5-6 years, have reported good treatment results for ophthalmopathy and no tumor development; they have also reported no difference in treatment efficacy between 10-fraction and 5-fraction irradiation. Furthermore, with the IMRT technique, The CTV encompassed the origins of insertions of the extra-ocular muscles and the retroorbital fatty spaces with the main bulk [2]. The lenses, globes, optic nerve, and lacrimal glands were zoned as organs at risk. A 2 mm concentric margin around the CTV was generated as the PTV. The 90% isodose line covers the PTV.

No complications related to retinopathy have been reported in recent studies. Notably, in addition to advancements in treatment equipment, retrospective studies have identified variations in treatment planning. One study comparing contouring in the retro-orbital space to the original contour found an overlap of only 68% [7]. Interestingly, there was no significant association between improvement in color plates, visual fields, and visual acuity based on the variation in dosages. The study concluded that without a standardized contouring protocol for thyroid eye disease, there was significant variation in target delineation. However, differences in dose to the anatomic retro-orbital space did not affect outcomes in the follow-up.

There are many detailed reports of radiation-induced meningiomas in the literature [8–11]. Current exceptions to the main causes, which are childhood treatment for tinea capitis and exposure to atomic explosions in Hiroshima and Nagasaki, are considered uncommon [8]. The most significant cause of concern today is medically induced radiation-induced meningiomas, due to advances in radiation side effect concepts and protection. One major cause may be the use of radiation for therapeutic purposes. Since meningiomas are the most commonly occurring tumors in the brain, it is crucial to differentiate between spontaneous and induced diseases. They usually afflict a younger population and are inherently more aggressive than the spontaneous type [10]. A literature review of case reports reveals the following characteristics: the meningioma is more aggressive with higher doses of radiation, and it develops more rapidly than the spontaneous type [9,11].

If radical excision cannot be achieved, stereotactic radiotherapy or stereotactic radiosurgery may be preferable to conventional radiotherapy. Radiosurgery is a significant treatment modality for radiation-induced meningiomas [12–15]. It is important to note that while a coherent number of cases have been reported in the past, some series included only a few grade II meningiomas [12,15], and some intentionally excluded grade II meningiomas [13,14]. Radiosurgery is an effective treatment option for grade I or radiologically-diagnosed radiation-induced meningiomas [13]. A sufficient dose of 12 Gy provides satisfactory control rates either after resection or as an alternative to resection. It is important to note that radiosurgery is not always a suitable treatment option and should be evaluated by a medical professional on a case-by-case basis. Adjuvant conventional conformal radiation is generally recommended following surgery or radiosurgery to treat viable tumor cells that remain along the dura and in brain parenchyma in patients with atypical (WHO grade II) meningiomas. Careful consideration should be given to initial doses and treatment areas when planning new therapeutic radiation. In the present case, not only the retro-orbital lesion but also outside of the orbital lesion including sphenoidal bone, temporal cortex, and temporal muscle had been irradiated in the past, and fractionated radiosurgery was performed.

### Dose tolerance of optic nerve

For radiotherapy of diseases near the optic nerve, it is recommended to limit the radiation dose to the optic nerve, but it is important to note that the radiation dose should be adjusted to individual patient characteristics and that close monitoring of the optic nerve during and after treatment is necessary [16–18]. To prevent optic neuropathy at a rate of less than 1%, a dose of 10 Gy in 1 fraction, 20 Gy in 3 fractions, or 25 Gy in 5 fractions is recommended [19]. These recommendations are based on an analysis of pooled data from 34 published studies, which included 1578 patients. Recent reports suggest that radiation therapy using 25 Gy/5 fractions with CyberKnife or 10 Gy/1 fraction with gamma knife has achieved good local control rate in the absence of optic nerve damage [16,17]. However, these reports are limited to a specific number of grade II meningioma cases. For more aggressive cases of grade II meningioma, an increased radiation dose of 15 Gy or higher in a single fraction is necessary to ensure tumor control. The use of single fractionation may increase the risk of optic neuropathy when compared to hypofractionation treatment [20]. It was necessary to administer an adequate dose of radiation to the margin of the sphenoid bone, where the tumor originated, to prevent recurrence. Therefore, a 5-fraction treatment plan delivering 30 Gy of radiation was decided as appropriate. The visual pathway was designated as the organ at risk during the planning process. The maximum dose to the optic nerve in this treatment would be less than 5 Gy, considering a recommended radiation dosage of 25 Gy in 5 fractions and the patient's prior exposure to 20 Gy of radiation. Executing the preferred approach posed challenges, but was necessary to administer a sufficient dose to the tumor, an average of 6.9 Gy and a maximum of 9.3 Gy for the optic nerve, which was thought to be an acceptable risk in the present case. It is important to monitor both long-term tumor control and the likelihood of developing radiation-induced ophthalmopathy in the future.

## Conclusion

The occurrence of radiation-induced meningiomas as a result of using radiation to treat malignant neoplasms is rare as a late side effect. Treatment using stereotactic radiotherapy or stereotactic radiosurgery is an option for recurrent radiation-induced menigiomas, or residual lesions after surgery, to provide better local control rates. The development of highly aggressive meningiomas due to radiation for benign disease is an unexpected but potential risk to the patient's quality of life. Long-term observation and early treatment with less invasive approaches are desirable.

## Patient consent

Consent was obtained by all participants in this study.
